# Morphology of Starch Particles along the Passage through the Gastrointestinal Tract in Laboratory Mice Fed Extruded and Pelleted Diets

**DOI:** 10.3390/ani12080952

**Published:** 2022-04-07

**Authors:** Jasmin Wenderlein, Ellen Kienzle, Reinhard K. Straubinger, Heidrun Schöl, Sebastian Ulrich, Linda Franziska Böswald

**Affiliations:** 1 Chair of Bacteriology and Mycology, Institute for Infectious Diseases and Zoonoses, Department of Veterinary Sciences, Faculty of Veterinary Medicine, LMU München, Veterinärstr. 13, 80539 München, Germany; jasmin.wenderlein@micro.vetmed.uni-muenchen.de (J.W.); r.straubinger@lmu.de (R.K.S.); s.ulrich@lmu.de (S.U.); 2 Chair of Animal Nutrition and Dietetics, Faculty of Veterinary Medicine, Ludwig-Maximilians-Universitat, 80539 München, Germany; kienzle@tiph.vetmed.uni-muenchen.de; 3 Institute of Veterinary Pathology, Faculty of Veterinary Medicine, Ludwig-Maximilians-Universitat München, Veterinärstr. 13, 80539 München, Germany; schoel@patho.vetmed.uni-muenchen.de

**Keywords:** amylase, carbohydrate metabolism, processing, laboratory animal diets, caecum fermentation

## Abstract

**Simple Summary:**

Starch is the main carbohydrate source in most lab mouse diets. Its properties are influenced by feed processing. This determines how easily accessible it is to enzymatic digestion in the gastrointestinal tract of animals. In previous studies we have shown that there are differences between pelleted and extruded forms of a maintenance diet fed to mice regarding digestibility and microbiome. To complement these findings, the present study presents a morphological study of the starch particles throughout the passage along the gastrointestinal tract of C57BL/6J mice fed either pellets or extrudate. Samples were stained with Lugol’s iodine and examined via stereomicroscope and scanning electron microscope. Starch granules in the pelleted diet are mostly intact and compact, thus autoenzymatic digestion in the small intestine is less efficient than in the more accessible starch granules from the extruded diet. For both diet forms, starch accumulation in the caecum was observed, suggesting selective retention of praecaecally undigested starch for microbial fermentation. These findings allow for unique insights in murine starch digestion that are important to understand the digestive physiology of this species.

**Abstract:**

Diet processing impacts on starch properties, such as the degree of starch gelatinization. This affects digestibility, as shown in laboratory mice fed either a pelleted or an extruded diet. In the present study, the morphology of starch particles throughout the digestive tract of mice was visualized. Thirty-two female C57BL/6J mice were used for a feeding trial. They were fed a commercial maintenance diet for laboratory mice, which was available in pelleted and extruded form, for seven weeks. The mice were sacrificed after the feeding period, and chyme samples were collected from five sites (stomach, anterior and posterior small intestine, caecum, colon). Samples of diets, chyme and faeces were analyzed via stereomicroscopy (stained with Lugol’s iodine) and scanning electron microscopy (SEM). The starch granules appeared more compact in the pelleted diet, showing first signs of degradation only in the small intestine. The caecum content of both diets group was intensively stained, particles as well as fluid phase, indicating that it contained mainly starch. The SEM pictures of caecum content showed abundant bacteria near starch particles. This suggests selective retention of prae-caecally undigested starch in the murine caecum, likely the site of microbial fermentation.

## 1. Introduction

Rodent species show a wide variety of adaptations to their respective diet spectrum. They are typically hindgut fermenters with a well-developed caecum [1], the complexity and size of which may be related to fermentative activity. Mice and rats are omnivorous rodents with a high amount of seeds in their natural diets. In captivity, especially in most laboratory animal husbandries, they are fed cereal-based diets with a high carbohydrate content [2]. Digestive strategies of herbivorous and omnivorous small hindgut fermenters are well described regarding fiber digestion [2,3]. However, the fate of starch in the murine gastrointestinal tract has not been investigated in detail.

Starch properties, especially the structure of the starch granules, differ between the botanical sources. The structure of the granules may limit digestibility [4], depending on granule size and the presence and number of pores in the waxy outer layer of the granunle [5], among other factors. Feed processing causes major changes in the starch structure that can lead to the partial or complete destruction of the starch granules, determining starch digestibility to a large extent. Gelatinization of starch occurs under the influence of heat and moisture, when starch granules swell and the crystalline structure degrades, making the molecule more accessible for digestion by amylase [4]. Extrusion uses heat, pressure, moisture and shearing forces to alter the structure of granules. The major force acting in the production of pellets is pressure. During this process, heat is only generated near the surface of the pellet. The starch gelatinization is generally lower in pellets than in extruded diets [4,6,7], which could also be shown for laboratory rodent feed [8]. In addition to the general process, individual differences in the specific adjustment of a manufacturing plant may influence the ultimate results of the feed processing. For optimum digestibility, the processing parameters need to be adjusted so that the granule structures are altered for enzymatic accessibility and re-crystallization does not occur at a high level [5]. 

According to its digestive characteristics, starch can be classified into rapidly vs. slowly digestible starch [9]. For example, starch from uncooked cereal sources is relatively slowly digestible [5]. In contrast, resistant starch is defined as the proportion of starch and its degradation products not absorbed in the small intestine [10]. There are several types of undigested starch passing the small intestine: RS (I) inaccessibility due to the original cell structure, e.g., unprocessed cereal grains; RS (II) starch granules resistant to enzymatic digestion, e.g., in potato or banana; RS (III) retrograded starch forming by polymer formation after processing during cooling and storage [5,9,10]. Retrograded starch precipitates in crystalline structures from the previously gelatinized starch molecules [9,11]. Types I–III of RS can be used at least in part for energy utilization in livestock animals [5].

An increase in starch gelatinization by heat processing increases energy digestibility and thus growth performance in pigs [12] and mice [13]. In horses, differences in starch digestibility have also been demonstrated using different starch sources and processing methods [14,15]. Furthermore, experiments on morphology and digestion of different types of starch in the different compartments of the gastrointestinal tract have been conducted in various species, e.g., horse [14], chicken [16], pigs [5] and ruminants [17]. In vitro models for visualization of starch degradation in different gastrointestinal sites have been set up [18,19]. Starch morphology as visible in the scanning electron microscope cannot exactly predict the starch digestibility in vivo but is valuable to understand and explain differences in digestibility [20]. 

The kinetics of starch degradation and digestion have been thoroughly investigated in livestock, as described above. There are species differences related to the functional morphology of the gastrointestinal tract. In mice, which are commonly used as laboratory animal, basic information on starch digestion is lacking. Because of the influence of starch digestion on energy utilization and intermediary metabolism [5,13], such knowledge is important for the use of mice as a model organism in research. We could already demonstrate the effect of pelleted and extruded diets on digestibility, body weight development [13] and the gastrointestinal microbiome [21] of laboratory mice. In this study, we aimed to describe the morphologic changes of a diet in extruded and pelleted form throughout its passage through the murine gastrointestinal tract. This can give insights into the site (small intestine vs. hindgut) and type of degradation (autoenzymatic digestion vs. alloenzymatic microbial fermentation) of starch. 

## 2. Materials and Methods

### 2.1. Animals and Diets

Ethical approval for the use of animals was obtained from the Ethics Committee of the Veterinary Faculty of the Ludwig-Maximilians-Universität München (reference no. 169-03-05-2019). Data on digestibility and microbiome from this study were reported previously [13,21].

For the trial, 32 eight-week-old C57BL/6J mice were used (Envigo RMS B.V., Horst, The Netherlands). Female mice were chosen so that pair-housing for the duration of the trial was possible. Mice were kept in a specific-pathogen-free facility (Techniplast isocages, Buguggiate, Italy, with Tigerino Crystals, Matina GmhH, Munich, Germany, as bedding material). Feed and water were available ad libitum. After adaptation to the surroundings, the mice were allocated randomly to two groups: Group PEL was fed a commercial maintenance diet for laboratory mice in pelleted form, group EXT received the same diet in extruded form. The diets were purchased from the same manufacturer and were labelled as identical maintenance diet for laboratory mice, available in pelleted and extruded form. Wheat was the major starch source in both diets.

A digestibility trial was conducted as described previously [13]. After seven weeks on the respective diets, the mice were sacrificed by cervical dislocation and dissected. Immediately after sacrifice, the mice were dissected and the gastrointestinal tract was removed from the body. The gastrointestinal tract was opened at defined sites (stomach, anterior and posterior small intestine, caecum, colon) and chyme samples were taken with sterile surgical instruments. 

### 2.2. Analyses

#### 2.2.1. Diet

Diet samples were ground, dried and analyzed in for gross energy (bomb calorimetry) and crude nutrient content (Weende analysis) by standard methods [22]. Dietary starch (polarimetric method according to directive (EG) 152/2009, annex III, L: 2009-02), total dietary fiber (TDF), soluble and insoluble fiber (based on AOAC method 991.43) as well as the degree of starch gelatinization (standard method according to VDLUFA III 7.2.6; 2012 [OL]) were also measured. Detailed information can also be found in the previous publications [13,21]. Several intact pellets and extruded kibbles were placed in a plastic petri dish (Sarstedt AG & Co. KG, Nümbrecht, Germany) and stained with 1-2 mL Lugol´s iodine (solution of 0.5 g iodine, 1 g potassium iodide in 600 mL distilled water) to detect starch (blue staining).

#### 2.2.2. Stereomicroscopy

Samples of both diets were crushed, stained with Lugol´s iodine and examined microscopically (Leica M60, Leica Microsystems GmbH, Wetzlar, Germany; possible magnification 6.3–40 ×). Of four animals per diet group, gastrointestinal content from different sites (stomach, anterior and posterior small intestine, caecum, colon) was collected from the fresh carcasses. Faeces was collected from the cages. 

**Figure 1:**
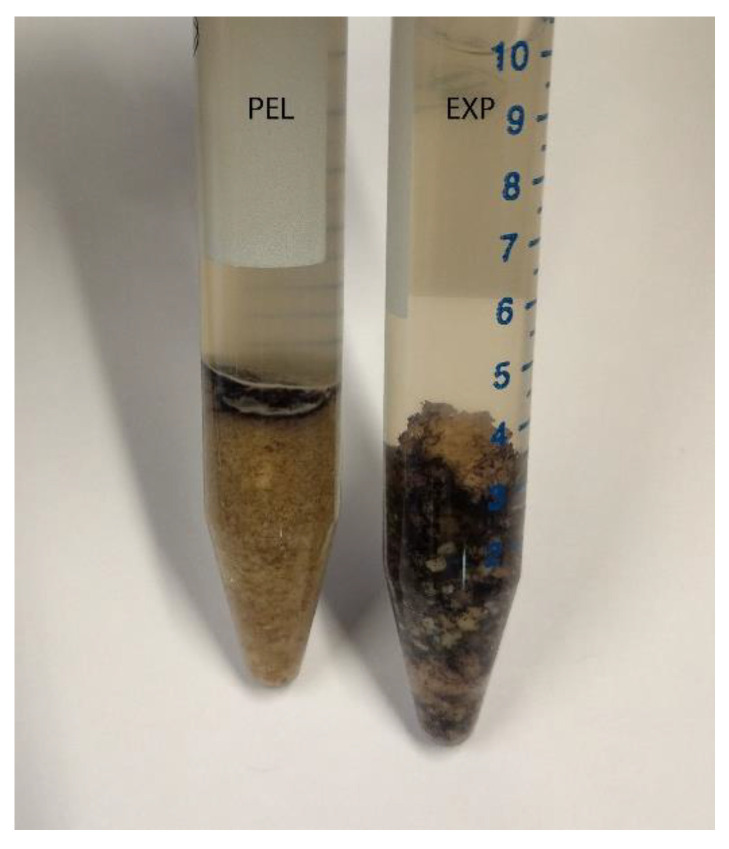
Centrifuged samples of the diets, showing a narrow blue band in PEL (on the left) and staining throughout the complete sediment in EXT (on the right).

The chyme and faeces samples were mixed with distilled water (1:5) for pH measurement (pH data reported previously [13]). After pH measurement, 1mL of Lugol´s iodine was added, and the samples were centrifuged (3× *g*, 5 min; Eppendorf 5810R, Eppendorf AG, Hamburg, Germany). The phase of sediment stained dark blue with Lugol´s iodine (Figure 1) was used to generate a subsample of approximately 0.25–0.5 mL (depending on the total amount) that was transferred to a plastic petri dish (Sarstedt AG & Co. KG, Nümbrecht, Germany) and examined microscopically. The routine magnifications we used were25 × and40 ×. For publication, the photographs were edited for light intensity in Adobe Photoshop^®^ (Adobe Inc., San Jose, CA, USA) and markers inserted in Adobe Illustrator^®^ (Adobe Inc., San Jose, CA, USA). 

#### 2.2.3. Scanning Electron Microscopy

Samples of diets, gastrointestinal content (sampling sites see above), and faeces were used for scanning electron microscopy (SEM; Zeiss DSM 950, Carl Zeiss Microscopy Deutschland GmbH, Oberkochen, Germany). From the diluted samples described in the stereomicroscopy section, 100 µL were transferred on a round glass slide attached to an aluminum sample holder with a carbon glue pad (both: Plano GmbH, Wetzlar, Germany). The sample was then sputtered using the Balzers Union SCD 040 (Oerlikon Balzers Coating Germany GmbH, Bingen, Germany) with the sample holder on 6 cm height. The slide was sputtered with gold-palladium coating with 15 mA for 310 sec until achieving a layer thickness of 12 nm. After this preparation, the sample holders were inserted into the SEM and examined with 10 kV. The SEM pictures were color corrected and adjusted for brightness and contrast (Adobe Photoshop^®^, Adobe Inc., San Jose, CA, USA). Marker symbols were added if necessary (Adobe Illustrator^®^, Adobe Inc., San Jose, CA, USA).

## 3. Results

### 3.1. Diets

Diet composition is summarized in Table 1. The diets differed in starch content and the degree of starch gelatinization, as was to be expected due to the respective method of processing. Diet EXT had a lower starch content (28% vs. 43%, as fed), but a higher degree of starch gelatinization than diet PEL (70% vs. 17% of starch). Corresponding to the difference in starch content, diet EXT contained more TDF than diet PEL (23 vs. 16% as fed), with a higher amount of insoluble fiber. 

### 3.2. Macroscopic Presentation

**Figure 2:**
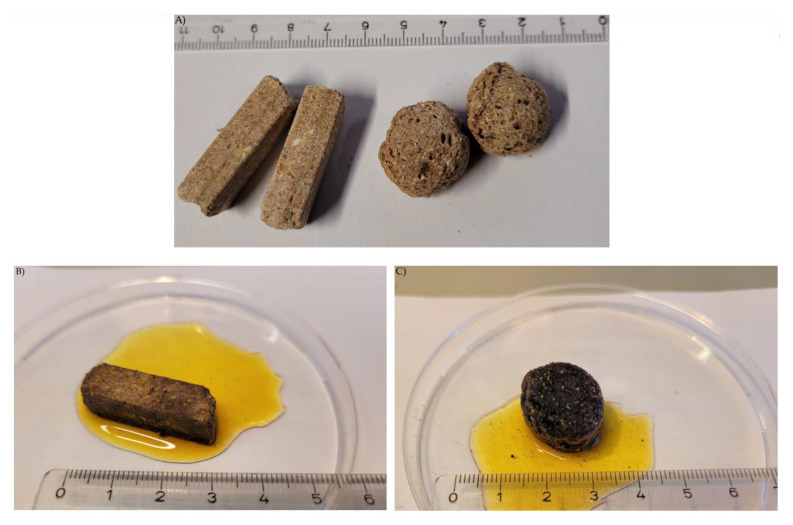
**Diets.** (**A**) Intact pellets (PEL) and extruded kibbles (EXT); (**B**) Intact pellet stained with Lugol´s iodine; (**C**) Intact extruded kibble stained with Lugol´s iodine.

Staining the intact pellets and extruded kibbles with Lugol´s iodine resulted in blue coloration, showing the presence and distribution of starch in the diets (Figure 2). The extruded kibble was stained faster and more intensively than the pelleted feed. In the fluid phase surrounding the kibble, stained particles of different sizes and shapes became visible. 

**Figure 3:**
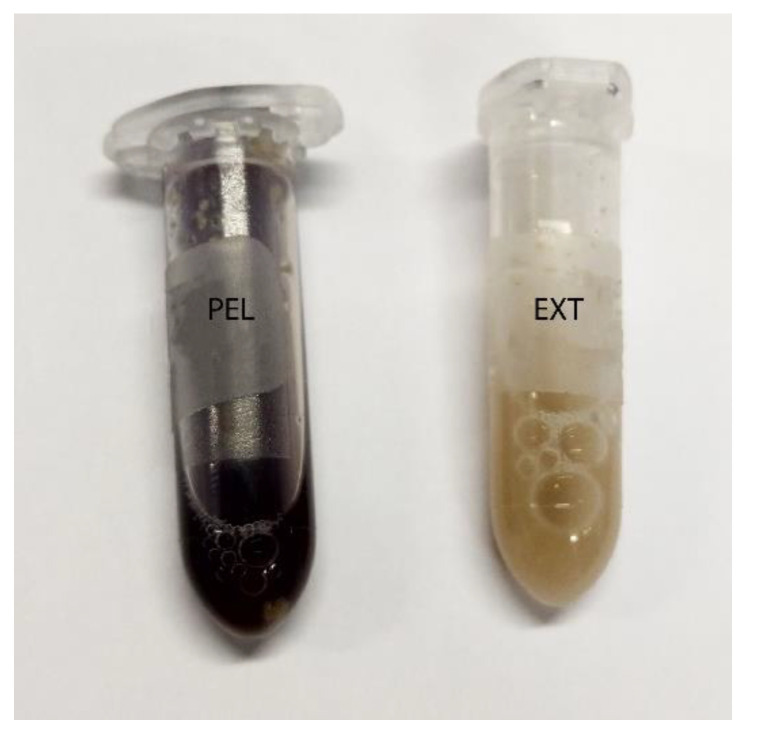
**Caecum content stained with Lugol´s iodine**. On the left is a sample of group PEL, stained entirely blue, and on the right a sample of group EXT.

After centrifugation, there were blue areas in the sediment in the diet and chyme samples from the stomach, small intestine and colon (example diet: Figure 1). Caecum content was an exception, since the complete sediment was stained. The caecum content of mice fed with diet PEL was stained completely in an intensive blue. The staining was less intensive and more of a violet shade in the samples from EXT mice (Figure 3). 

### 3.3. Stereomicroscopy

Stereomicroscopy images show starch particles stained by Lugol´s iodine in blue, enabling a qualitative description. The figures presented for publication were chosen representative for the respective sampling site. 

**Figure 4:**
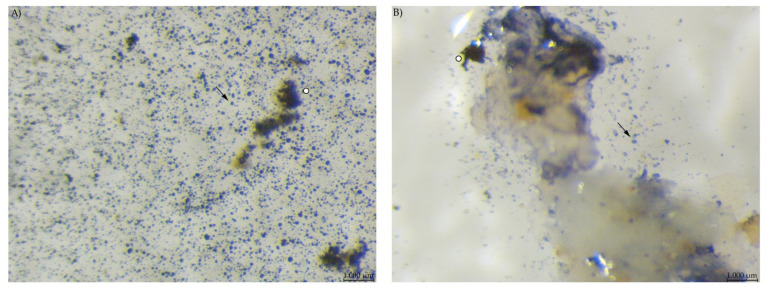
Diet samples stained with Lugol’s iodine. (**A**) PEL with a high number of small, round stained starch particles; (**B**) EXT with less compact stained particles, larger fragments and starch in the soluble fraction (stereomicroscopy, 40 ×; ○ marking larger fragments of starch, ↓ marking small round particles).

Microscopic evaluation of the diets showed a high number of small, round stained particles in diet PEL. Diet EXT contained stained particles of irregular shape and also a bluish stain in the fluid phase (Figure 4). 

**Figure 5:**
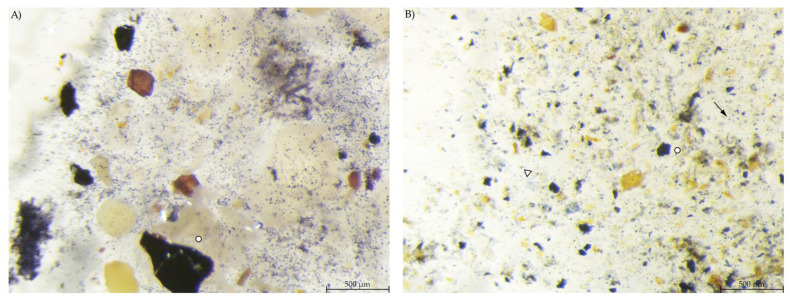
Stomach content samples stained with Lugol’s iodine. (**A**) PEL with compact, round stained starch particles; (**B**) EXT with round and irregular stained particles and a blue haze in the fluid phase (stereomicroscopy, 25 ×; ○ marking larger starch fragments, ↓ marking small round particles, ∆ marking blue haze).

In the stomach content, the blue-stained particles were similar to those in the respective diet regarding amount and shape. EXT stomach samples showed few small, round stained particles, large stained particles and a bluish haze around the particles (Figure 5). 

**Figure 6:**
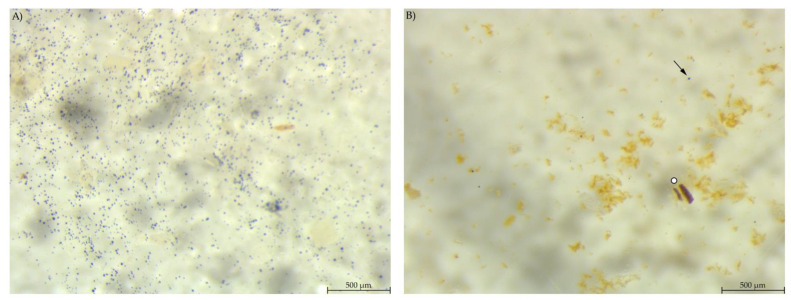
Samples from the anterior small intestine stained with Lugol’s iodine. (**A**) PEL showing a higher amount of small, round stained particles than (**B**) EXT (stereomicroscopy, 25 ×; ○ marking larger fragments of starch, ↓ marking small round particles in **B**).

Chyme from the anterior small intestine of group EXT mice contained a much lower number of stained particles visible than in the stomach samples (Figure 6). In group PEL, the content of the anterior small intestine still showed a high number of small, round starch particles. However, unlike in the stomach, there were no large stained particles. The picture was much the same in the posterior small intestine, with fewer starch particles overall than in the anterior small intestinal samples. 

**Figure 7:**
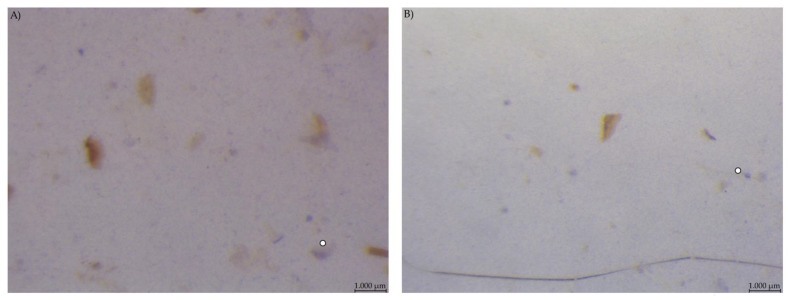
Caecum content stained with Lugol’s iodine. (**A**) PEL and (**B**) EXT, both with stained streaks dominating the picture (stereomicroscopy, 25 ×; ○ marking larger fragments of starch).

Caecum content samples from both diet groups were difficult to get into focus using the light microscope (Figure 7). The fluid phase was stained in streaks so that particular shapes were difficult to identify. 

**Figure 8:**
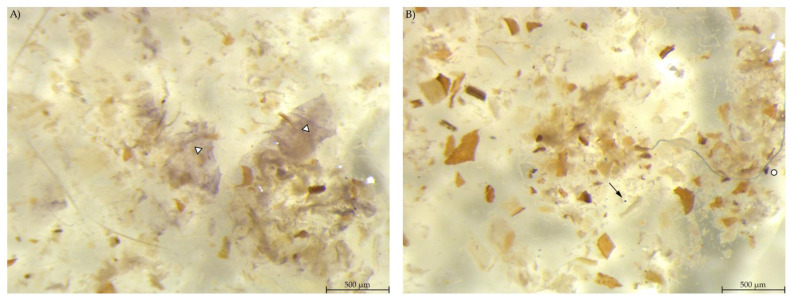
Colon content stained with Lugol’s iodine. (**A**) PEL with smears of stained starch; (**B**) EXT with hardly any stained particles visible (stereomicroscopy, 25 ×; ○ marking larger fragments of starch, ↓ marking small round particles, ∆ marking blue haze).

In the colon content, a smaller amount of stained material was found compared to the other gastrointestinal samples (Figure 8). PEL samples showed areas with blue stained smears, similar to the stomach samples from group EXT. The colon content of EXT mice did hardly contain any stained material. 

Faecal samples also did not contain many starch particles. The single particles that were stained in those samples were of irregular shape, but relatively compact and without a smear or haze in the fluid phase (Figure S1). There was no marked difference between the diet groups. 

### 3.4. Scanning Electron Microscopy

The focus of the SEM visualization was on the starch particles in the different samples. For each sample or sampling site, a representative figure was chosen for publication. 

**Figure 9:**
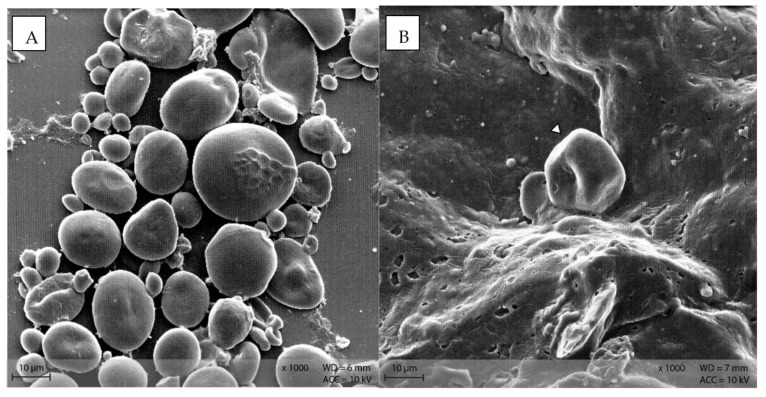
Starch granules in the diets (**A**) PEL as round, compact granules of different size, and (**B**) EXT as irregularly shaped particle (Scanning electron microscopy; ∆ marking irregularly shaped starch particles in **B**).

In **diet** PEL, the starch particles were visible as compact, round structures (Figure 9A), while in diet EXT, the granules were rather irregularly shaped with a visible indentation in the middle of a formerly round granule (Figure 9B). 

**Figure 10:**
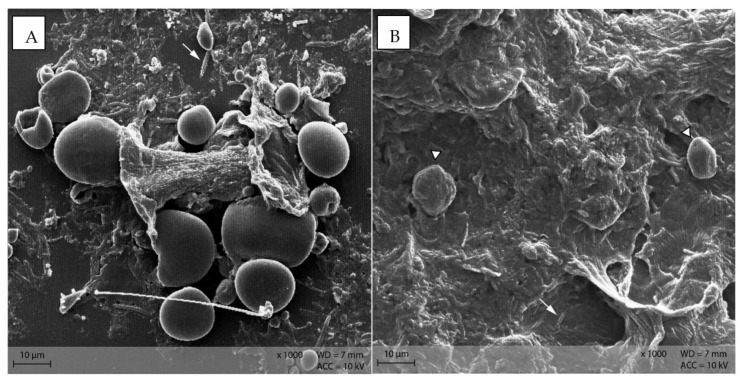
Starch particles in the stomach content of group (**A**) PEL, and (**B**) EXT; note the rod-shaped bacteria in the surroundings of the starch particles. (Scanning electron microscopy; ↓ indicating bacteria, ∆ marking irregularly shaped starch particles in **B**).

Stomach content samples showed starch particles shaped similarly to the respective diet sample (Figure 10). In both groups, rod-like shapes were visible in the stomach content samples.

**Figure 11:**
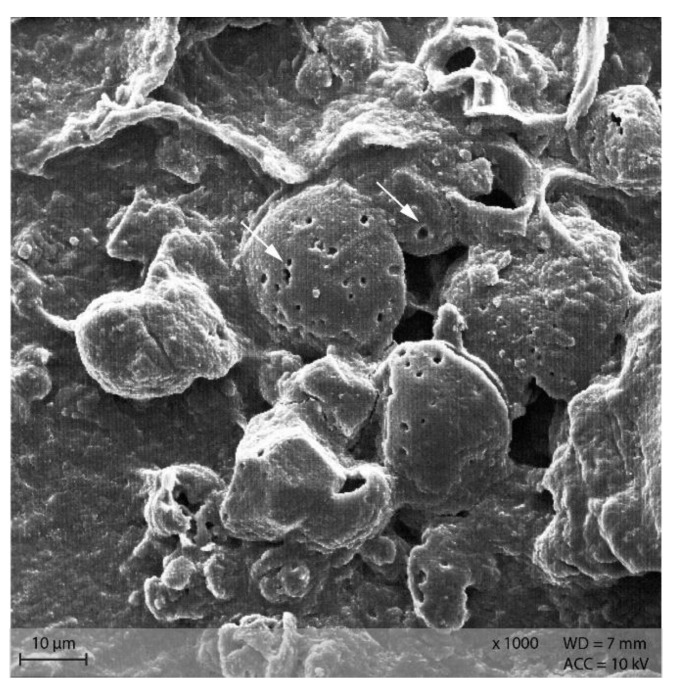
Starch granules with small pinholes, indicating the beginning of enzymatic degradation in the small intestine. Example from diet PEL (Scanning electron microscopy; ↓ indicating pinholes).

**Figure 12:**
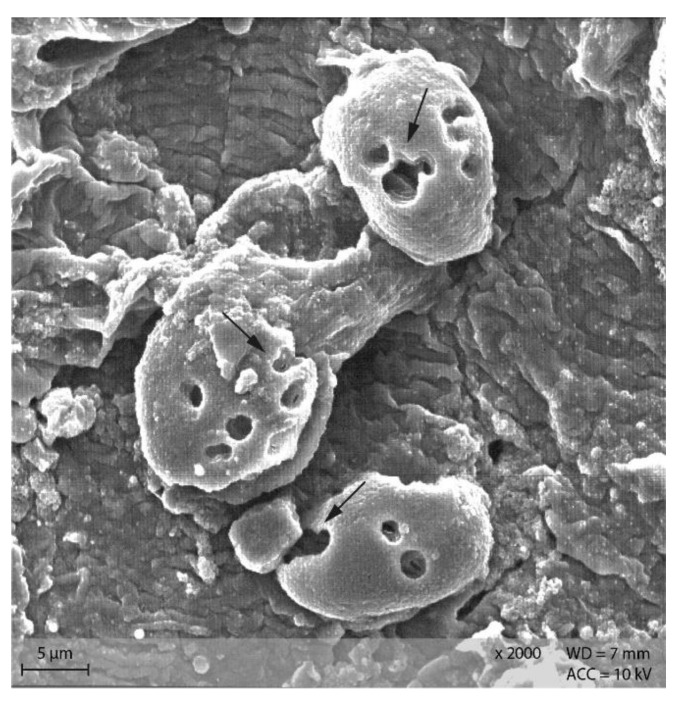
Starch granules from the posterior small intestine, showing large and partly confluencing holes (indicated by ↓). Example from diet PEL, scanning electron microscopy.

Starch particles in the anterior small intestine of both diets showed the beginning of disintegration with small pinholes (Figure 11). In the posterior small intestine, the holes were larger and the granules showed a higher degree of disintegration than in the anterior small intestine content (Figure 12).

**Figure 13:**
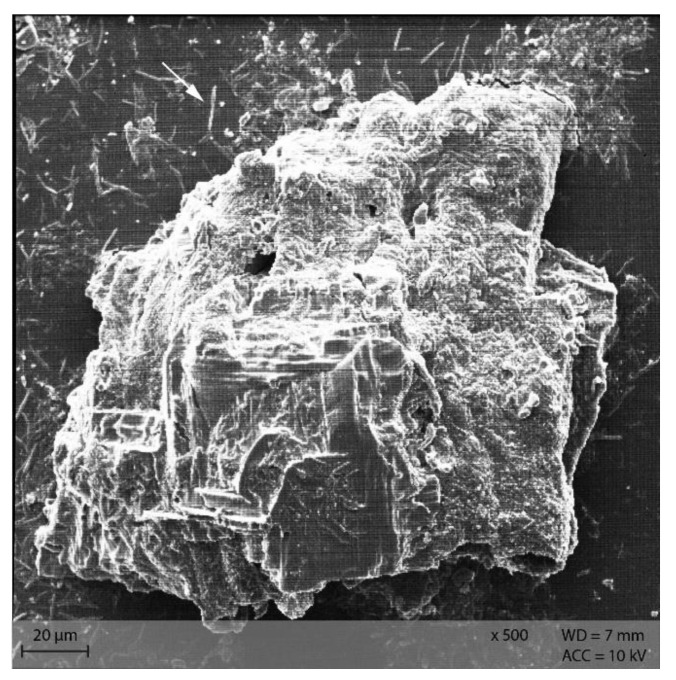
Caecum content sample from diet group EXT showing a large, crystalline shape (Scanning electron microscopy, ↓ indicating bacteria in the background).

**Figure 14:**
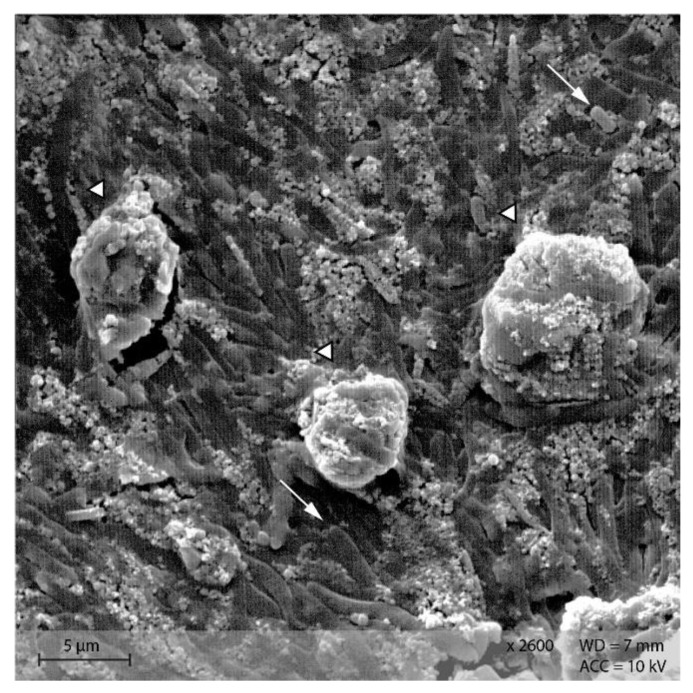
Starch granules in the course of degradation, surrounded by bacteria and small fragments. Sample of caecum content from group PEL (Scanning electron microscopy, ↓ indicating bacteria, ∆ marking the starch granules).

In the caecum content of both diet groups, crystalline rectangular structures were found (Figure 13). Figure 14 shows PEL caecum content with starch granules in degradation, surrounded by rod-like and coccoid structures (bacteria), as well as small irregular shaped “fragments” of the starch particles. Figure S2 highlights the presence of these fragments and bacteria.

## 4. Discussion

Stereomicroscopy and SEM images show the morphology of starch particles, indicating differences between the diets and changes throughout the gastrointestinal tract. The shape of the starch in the pelleted diet was typical for cereal starch in a processed feed [14,15]. There was a difference in starch morphology between the pelleted and extruded diets, with a higher degree of disintegration of the starch granules in diet EXT (Figure 9B). This corresponds with the higher degree of starch gelatinization in this diet and the processing-related effects [6,7]. However, the starch granules are still intact and apparently not damaged by processing in diet PEL. In addition, the blue smear in the fluid phase of the diet samples prepared for stereomicroscopy indicated a higher amount of soluble starch in the extruded diet (Figure 4B). In this study, commercial diets for laboratory mice were used for standardization purposes. The “natural” diet of mice in the wild may contain a different carbohydrate pattern and lack processing. Thus, in free-ranging mice the botanical source of starch is likely to have the most influence on starch digestibility instead of diet processing type. Nonetheless, the present results show basic kinetics of starch digestion in the murine gastrointestinal tract that are valuable not only for laboratory and pet animals.

Stomach content showed a similar morphology of starch particles as in the diets. Bacteria were present as depicted on the SEM images of stomach content (Figure 10). This is consistent with the results from 16S rRNA sequencing of the microbiome: Stomach content had comparable bacterial richness (α-diversity) as caecum and colon content, with Muribaculaceae, Lachnospiraceae and Lactobacillaceae as the most abundant bacterial families [21]. The murine stomach is divided into the non-glandular proventriculus, or forestomach, and the glandular ventriculus [23,24]. The non-glandular forestomach may be the first site of microbial fermentation as it is known for other species [1,25]. From the samples examined in this study, no differentiation of stomach locations was conducted due to the overall small sample size of murine stomach content. Thus, it is not possible to ascertain whether the bacteria observed in the SEM pictures and 16S rRNA sequencing are definitely located in the non-glandular forestomach. Further investigations are warranted to determine the fermentative processes in the murine stomach.

The SEM pictures from small intestinal content displayed the beginning enzymatic digestion of the starch granules by pancreatic amylase. Pinholes appeared as a first sign of damage to the granules [14], especially in the anterior small intestine of mice in the PEL diet group (Figure 11), with larger openings in the granules in the posterior large intestine (Figure 12). These observations correspond with the findings in horses fed maize [14]. Stereomicroscopy of small intestinal content also showed differences between the diet groups: In the anterior small intestine content of mice fed the EXT diet, only a small number of stained starch particles was visible, while in diet PEL, stained small round starch particles were present in abundance. This implies a difference in small intestinal digestibility of starch between the diets, with a higher autoenzymatic digestibility of the extruded starch (higher degree of gelatinization). However, in the apparent total tract digestibility (ATTD), this difference is not visible. The ATTD is calculated via nutrient content in diet and faeces, so it does not contain information about the location of degradation in the gastrointestinal tract (small intestine vs. hindgut). In the digestibility trial of this study [13], ATTD of starch was 99% for both diets with no significant difference. Determining the prae-caecal (small intestinal) digestibility of a nutrient would not be feasible in mice, because the amount of chyme in the small intestine is much less than what is needed for analysis. Thus, the morphologic investigations provide unique insights into the digestive process of starch throughout the gastrointestinal tract of mice.

Addition of Lugol´s iodine lead to complete blue or blue-violet staining of the caecum content samples, indicating a high abundance of starch in this compartment (Figure 3). Every particle seen in the stereomicroscope was stained. This may suggest selective retention of prae-caecally undigested starch into the caecum, comparable with the selective retention and separation mechanisms of other small mammals. In lagomorphs, possums, voles and lemmings, for example, there is a “wash-back” mechanism of retrograde peristalsis in the colon, transporting soluble particles back into the caecum [26]. For some other rodent species, separation mechanisms of soluble and solid particles in the colon without retrograde movement are described [26]. It seems likely that the caecum is the site of microbial fermentation of previously undigested starch, which has disintegrated from the granule structure. The SEM images of caecum content show rod-like shapes encircling the starch particles (Figure 13 and Figure 14). They may represent rod bacteria, which would match the families Lachnospiraceae, Muribaculaceae, and Lactobacillaceae comprising the core microbiome in the caecum content of the mice in this study [21]. Species from all three bacterial families are able to ferment plant polysaccharides, including starch [27,28,29]. In close vicinity to the larger starch particles and the bacteria, there were smaller, irregularly shaped particles in high abundance (Figure 14 and Figure S2). These may be the fragments of starch granule disintegration, which appear as a blueish smear in the stereomicroscopic assessment (Figure 7). The high presence of rod-shaped bacteria near partly degraded starch particles in the caecum content supports the idea of selective starch retention for microbial fermentation in the murine caecum. The crystalline structures visible in the SEM (Figure 13) are likely to be retrograded starch that was not digested enzymatically in the small intestine [30,31].

In the colon content samples, the abundance of starch particles was much lower than in the previous gastrointestinal sites. However, the samples from the PEL group showed diffuse staining of starch in the soluble phase and larger, irregularly shaped particles. This suggests that there was a part of the starch escaping both auto-enzymatic digestion in the small intestine and microbial fermentation in the caecum. Either the larger starch particles were not retained in the caecum for fermentation, as speculated above for the main amount of starch, or it may be resistant starch not even degraded by the microbes.

Faeces samples did hardly contain any stained starch particles at all. This is in accordance with the ≈99% apparent total tract digestibility of starch in both diet groups. Starch digestion and fermentation seem to take place in the small intestine and the caecum, utilizing nearly the entire amount ingested. The present morphologic study shows differences in the ratio of these two processes due to diet processing.

## 5. Conclusions

The morphology of starch particles in the diet differs according to diet processing. This difference influences the morphological changes of starch during the digestive process in mice, as observed via stereomicroscope and SEM. The number of starch particles in the posterior small intestine and the caecum suggests differences in small intestinal starch digestibility according to processing. The accumulation of starch, combined with the presence of bacteria, in the caecum suggests the selective retention of starch for microbial fermentation in the murine caecum.

## Figures and Tables

**Table 1:** Composition of the pelleted (PEL) and extruded (EXT) test diets (as fed basis).

	Diet PEL	Diet EXT
Gross energy (MJ/kg)	16.6	17.3
Dry matter (%)	89.5	88.5
Crude protein (%)	18.7	22.5
Crude fat (%)	2.5	5.1
Crude ash (%)	4.8	6.1
Crude fiber (%)	5.2	5.8
Starch (%)	43.0	28.0
Degree of starch gelatinization (% of starch)	17.0	70.0
Total dietary fiber (%)	15.9	23.3
Soluble dietary fiber (%)	2.0	3.9
Insoluble dietary fiber (%)	13.9	19.4

## Data Availability

All relevant data of this experiment is presented in the manuscript. Further information is available on request from the corresponding author.

## Supplementary Materials

The following supporting information can be downloaded at: https://www.mdpi.com/article/10.3390/ani12080952/s1, Figure S1: Faeces stained with Lugol´s iodine; Figure S2: Starch degradation in the caecum of group P.
