# Supplementary material for: Morphology of Starch Particles along the Passage through the Gastrointestinal Tract in Laboratory Mice Fed Extruded and Pelleted Diets
Source: Animals (Basel). 2022 Apr 7;12(8):952. doi: 10.3390/ani12080952 (PMC9032392; doi:10.3390/ani12080952)
Supplement: Supplementary file 1 [file animals-12-00952-s001.zip › animals-1629369-supplementary.pdf]

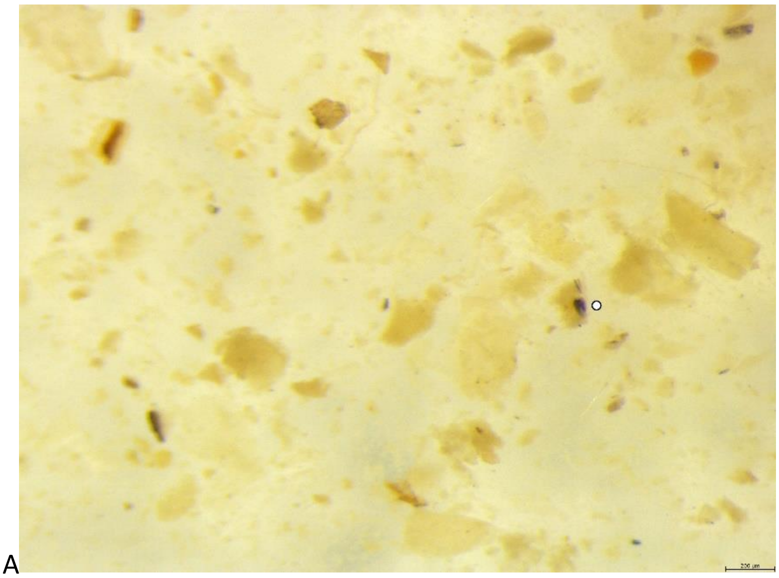
A

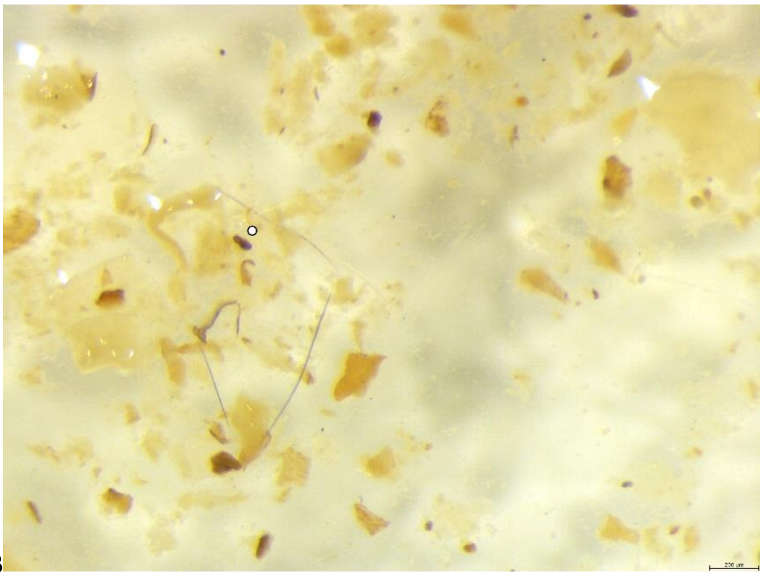
B

Figure S1. Faeces stained with Lugol’s iodine. (**A**) PEL and (**B**) EXT with single blue particles (stereomicroscopy, 25 ×; ○ marking larger fragments of starch).

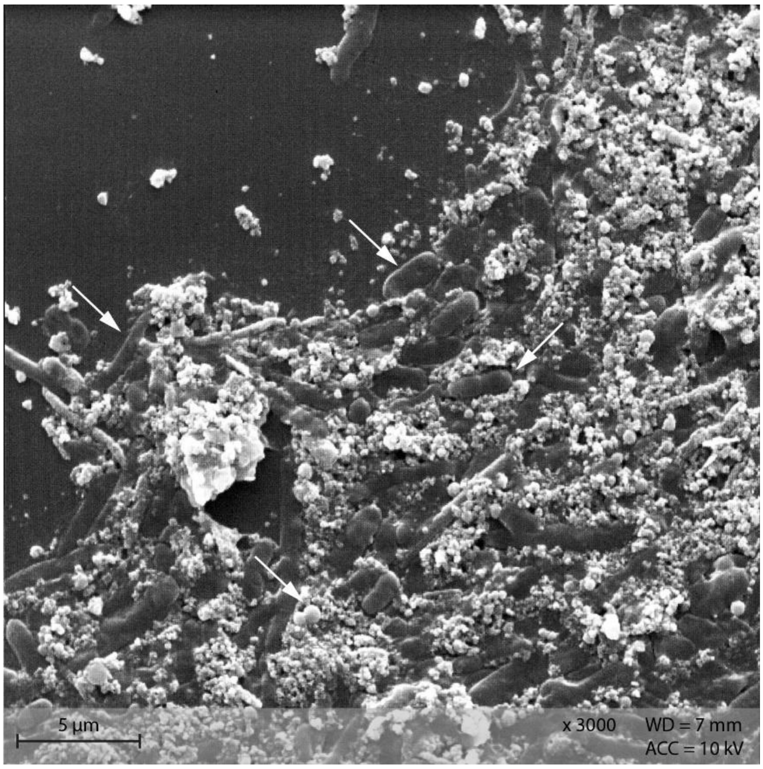

Figure S2. Starch degradation in the caecum of group P, with smaller starch fragments and a high number of bacteria visible (Scanning electron microscopy, ↓ indicating bacteria).
